# ENGAGING THE PAST FOR THE FUTURE: APPLYING ANTIRACIST PEDAGOGY TO HEALTH DISPARITIES IN LATER LIFE

**DOI:** 10.1093/geroni/igae098.3110

**Published:** 2024-12-31

**Authors:** Rona Karasik, Kyoko Kishimoto, Isaac Asirifi Boateng, Camryn Hafner, Claudia Burgos Zuniga

**Affiliations:** Saint Cloud State University, Saint Cloud, Minnesota, United States; Saint Cloud State University, Saint Cloud, Minnesota, United States; Saint Cloud State University, Saint Cloud, Minnesota, United States; Bournewood Health Systems, Somerville, Massachusetts, United States; Saint Cloud State University, Saint Cloud, Minnesota, United States

## Abstract

Pervasive health disparities among racial groups are well documented throughout the life course and across generations. The continued persistence of these disparities, however, suggests that simply recognizing their presence is insufficient for preparing future gerontologists, care-providers, and policymakers to address them. How can educators help gerontology students not only acknowledge the existence and impact of such disparities, but also to consider their root causes - an essential step toward effecting social change? Moreover, how can we prepare gerontology educators to guide students through this exploration? Building on previous content compilations regarding housing (Kishimoto & Karasik, 2018) and retirement (Karasik & Kishimoto, 2022), and integrating emerging research and educational approaches from fields such as Public Health (e.g., Braveman et al., 2022; Jones et al., 2018; Yearby et al., 2022), this presentation showcases newly developed/adapted educational materials and activities geared toward facilitating gerontology students’ exploration of racial health disparities from an antiracist perspective. Central to the provided lesson plans is an annotated timeline pairing key historical events with health care related policies and programs in the U.S., as well as multi-media resources on related topic areas. Given the current political climate (e.g., the dual influences of Black Lives Matter and anti-DEI legislation) and the varying extent of students’ (both domestic and international) prior understanding of US history, strategies for supporting productive classroom discussions on racial health disparities in later life are also shared from both faculty and student perspectives.

